# Formation of miRNA Nanoprobes—Conjugation Approaches Leading to the Functionalization

**DOI:** 10.3390/molecules27238428

**Published:** 2022-12-02

**Authors:** Iveta Vilímová, Katel Hervé-Aubert, Igor Chourpa

**Affiliations:** EA6295 Nanomédicaments et Nanosondes, Université de Tours, 37200 Tours, France

**Keywords:** miRNA, conjugation strategy, covalent bonding, non-covalent bonding, nanomaterial

## Abstract

Recently, microRNAs (miRNA) captured the interest as novel diagnostic and prognostic biomarkers, with their potential for early indication of numerous pathologies. Since miRNA is a short, non-coding RNA sequence, the sensitivity and selectivity of their detection remain a cornerstone of scientific research. As such, methods based on nanomaterials have emerged in hopes of developing fast and facile approaches. At the core of the detection method based on nanotechnology lie nanoprobes and other functionalized nanomaterials. Since miRNA sensing and detection are generally rooted in the capture of target miRNA with the complementary sequence of oligonucleotides, the sequence needs to be attached to the nanomaterial with a specific conjugation strategy. As each nanomaterial has its unique properties, and each conjugation approach presents its drawbacks and advantages, this review offers a condensed overview of the conjugation approaches in nanomaterial-based miRNA sensing. Starting with a brief recapitulation of specific properties and characteristics of nanomaterials that can be used as a substrate, the focus is then centered on covalent and non-covalent bonding chemistry, leading to the functionalization of the nanomaterials, which are the most commonly used in miRNA sensing methods.

## 1. Introduction

The detection of miRNAs (short, non-coding RNA sequences of approximately 19 to 25 nucleotides) has tremendous potential for early diagnosis of varying disorders, most notably life-threatening and unpredictable diseases such as cancer. Their function as circulating biomarkers is however hindered by their sparseness in body fluids, small size, and minor differences between the types of miRNAs. Thus, their detection in a short time with high accuracy in biological samples remains a challenge. Body fluids affect large spectra of detecting systems due to their high viscosity, the pH influence, the presence of other interfering biomolecules, and as previously mentioned, low amounts of target miRNA [1].

The miRNA expression spectrum in respective body fluids can differ not only depending on the disease type and pathological conditions but also on other aspects related to the patient (e.g., medication, diet, age, etc.) The body fluids in which miRNAs are detected can be obtained by either invasive or non-invasive procedures. Currently, the research is more often focused on the detection in body fluids obtained by non-invasive or weakly invasive means, such as urine, whole blood, and serum. For the miRNA capture, the body fluid is typically mixed with nanoprobe suspension [1,2].

Methods and approaches of miRNA detection based on nanotechnologies offer an alternative path to a faster and less complicated detection process, nevertheless also with specific challenges, including reproducibility, standardization, optimization, normalization, and data processing. The techniques vary in utilized material (organic, inorganic, or hybrid), nanostructures (nanorods, nanowires, nanosheets, etc.), and the strategy of the complementary DNA application (hairpin conjugates, molecular beacons, catalytic self-assembly, spherical nucleic acids, etc.) [3,4,5].

As such, hybrid NPs are particularly interesting since they combine the unique physical properties of the inorganic core (especially useful for their detection/quantification) with the biocompatibility/biospecificity of the organic shell.

After the selection of the nanomaterial as the substrate, for example, for the nanoprobe, the next step is the functionalization via an appropriate conjugation strategy, generally in the form of the attachment of an oligonucleotide sequence. The cornerstone of miRNA targeting approaches is antisense technology based on hybridizing the target miRNA with a complementary oligonucleotide sequence, leading to the formation of a duplex with higher stability. Usually, a complementary miRNA of the same length as the target miRNA is used, since such molecules are widely commercially available along with a wide range of modifications. Other possibilities, such as single- or double-stranded DNA, various hairpin probes, and molecular beacons, depend on the chosen approach and subsequent detection technique. The form of the target miRNA depends on the chosen media of miRNA detection—for validation of a method in model solutions, it is possible to use commercially produced sequences mimicking the naturally occurring miRNA, whose detection in biofluids can be hindered by other biomolecules and proteins present [6].

**Figure 1:**
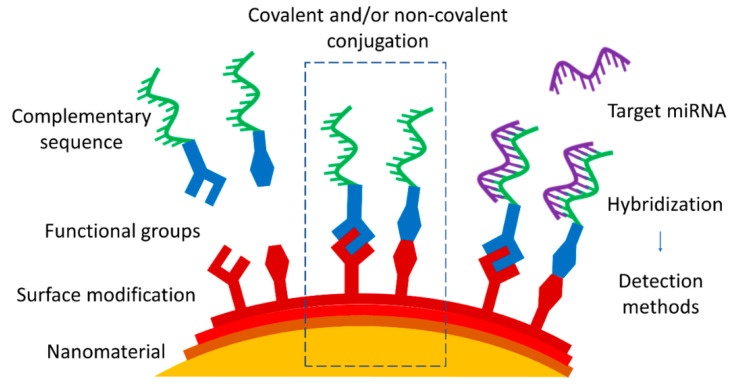
Position of the focus of this review, i.e., approaches of nanomaterial functionalization by covalent or non-covalent conjugation with complementary miRNA or DNA sequence as a part of the strategy of miRNA nanoprobe development.

The preparation of the functionalized material can be, at times, less scrutinized on the road to miRNA detection, the reports often understandably focused on achieving the lowest detection limits [7]. The review articles regarding miRNA detection methods analyze new developments and novel experimental techniques [5,8] or are devoted to some particular nanomaterial [9] or functionalized ligands [10]. However, to the best of our knowledge, none of those reviews is focused on the general strategy of functionalization and on its validation, in particular on the important details of the experimental protocol. In view of this situation, we suggest this review as an attempt to gain a deeper insight in the conjugation strategies and methods (Figure 1). In particular, this review is focused on the most common conjugation approaches applied to connect the nanomaterial and complementary sequences that are used to target specific miRNA. Since each nanomaterial presents its own unique properties and characteristics beyond those common to all, they are briefly outlined in the first part, along with the suggestion of potential characterization techniques. The following section describes the choice of conjugation approaches, divided into covalent and non-covalent ones, with drawbacks and advantages presented for each.

## 2. Nanomaterials Used as Substrates

All nanomaterials mentioned in this brief overview have high surface energy and a high surface-to-volume ratio, leading to a higher catalytical activity. Their more specific properties vary depending on the nanomaterial, which oftentimes leads to a combination of two or more types of nanomaterials to take better advantage of their strengths (Table 1).

### 2.1. Gold Nanoparticles

Gold nanoparticles (AuNPs) have excellent catalytic and electrical properties and unique optical properties, which are extensively studied for signal amplification with the aim of reaching highly sensitive biosensing. Their optical properties are rooted in the localized surface plasmon resonance (LSPR) phenomenon, and additionally, the plasmonic surface can quench the fluorescence. With the increase of their size and/or upon aggregation of the AuNPs in aqueous suspensions (colloids), their LSPR band shifts are detected by means of a UV-Vis spectrometer or seen with a naked eye as a color change [11].

AuNPs are biocompatible due to their chemical stability in biological fluids. Possible toxicity concerns are connected to their long-term retention in the body. On the other hand, AuNPs can be easily modified with miRNA molecules, particularly due to the gold surface affinity to the thiol group [12]. Used as electrochemical or optical sensors in fluorescence, surface-enhanced Raman scattering (SERS), surface plasmon resonance (SPR), or colorimetry-based detection approaches, AuNPs can either be dispersed in the aqueous media [13] or immobilized on solid support [14]. The enhancement of AuNPs is widely used in the applications of biosensors in miRNA detection, achieving higher selectivity and sensitivity starting from sub-nanomolar levels [11].

### 2.2. Silver Nanoparticles

Compared with AuNPs, the LSPR band of silver nanoparticles (AgNPs) has a shorter position (approximately 400 nm instead of 535 nm). Nevertheless, both the monodispersity and reproducibility are more difficult to control with AgNPs compared with AuNPs. This leads to a frequent combination of AgNPs with other metals, such as gold, for example, in the form of core–shell nanoparticles [15,16,17].

AgNPs have found application as sensors in electrochemical and optical detection due to their high extinction coefficient, high scattering-to-extinction ratio, and high field enhancement. AgNPs show distinct amplified signals [18] along with strong Raman and fluorescence enhancement. Successful approaches are based on plasmonic properties of AgNPs implemented in platforms applying SERS [19], LSPR [20], or fluorescence readouts for detecting miRNA [21]. Similar to other metal nanoparticles, AgNPs also need a compatible organic coating before their use in biological systems to positively influence their stability and possible cytotoxicity [22,23].

### 2.3. Magnetic Nanoparticles

The term “magnetic nanoparticles” (MNPs) generally encompasses metal (e.g., iron, nickel platinum, and cobalt), metal oxide (e.g., iron oxides Fe_3_O_4_, γ-Fe_2_O_3_, and ferrites), and metal alloy NPs (e.g., FePt and FeCo). MNPs present several advantages enabling their application in biomedicine, since after proper modification, MNPs are non-toxic and biocompatible and can be utilized as nanovectors for specific targeting. Their magnetic properties allow the use of magnetic separation for the binding and detection of biomolecules [24,25]. MNPs are often combined with other nanomaterial types to unite the specific advantages [26].

Considering that miRNAs are generally present in very low concentrations in body fluids, the possibility to reconcentrate the miRNAs captured by MNPs via magnetic separation or extraction presents an enticing enhancement in the detection process. The miRNA extraction from a larger volume sample also represents insight into a larger population of the molecules and provides simpler handling procedures. However, the magnetic separation can be hampered if the viscous drag of body fluids overwhelms the magnetophoretic force [27].

### 2.4. Quantum Dots

Quantum dots (QDs) are luminescent semiconductor nanocrystals with the possibility to tune the maximum wavelength of their light emission spectra. Stable QDs can be obtained by an easy one-pot and/or one-step synthesis directly in a water medium [28]. Aqueous suspensions of QDs have a high photoluminescent quantum yield and resistance to photobleaching [29]. These qualities prompted their advancing application as optical, electrochemical, and chemiluminescent biosensors [4].

Due to high surface reactivity, QDs can serve as a nanoscale scaffold for further functionalization with common conjugation chemistry.

All in all, QDs are useful in miRNA detection due to their strong fluorescence provided by high quantum yield, narrow emission, and broad absorption spectra which provide multicolor labels with one light source and a strongly active surface for conjugations. Interactions on the surface of QDs lead to the activation or quenching of the fluorescence signal, allowing miRNA detection in complex media, such as body fluids.

### 2.5. Carbon Nanomaterials

Various carbon nanomaterials offer different characteristics (higher surface area, biocompatibility, and non-toxicity) useful for biosensing. Generally, carbon nanoparticles have strong and adjustable photoluminescence [30].

Carbon nanomaterials for miRNA detection can be in the form of carbon nanoparticles [31], carbon nanotubes [32], nanofibers [33], quantum dots [34], fullerenes [35], graphene nanosheets [36], and graphene oxide [37].

The good electrical conductivity and sensitivity of carbon nanomaterials are applied for the design and construction of electrochemical biosensors, particularly for electrode surface modification and for the preparation of modified electrodes [30,38,39,40].

**Table 1:** Types of nanomaterials used as substrates for miRNA nanoprobes.

Nanomaterial	Advantages	Disadvantages	References
Gold	easy synthesis and modification chemical stability affinity to a variety of ligands plasmonic properties	poor degradability possible long-term toxicity	[11,14,41]
Silver	electrochemical detection plasmonic properties stronger than with gold	poor degradability possible cytotoxicity	[18,20,42]
Magnetic	superparamagnetism magnet-based purification andseparation	in larger size prone to magnetic aggregation	[1,43,44]
Quantum dots	strong and stable fluorescence high surface reactivity high electron transfer	stabilizers and surfactants needed on the surface	[28,45,46]
Carbon	widely available carbon sources fluorescence properties electrocatalytic activity	few in vivo studies performed	[30,37,47]

### 2.6. Characterization of Properties of the Nanomaterial before and after Conjugations

The determination of the physico-chemical properties of the chosen nanomaterial goes hand in hand with the need for monitoring their changes, both before and after the successful conjugation of a biomolecule. Some characterization techniques are more popular than others; however, as a rule, there is usually a combination of two or more methods used to cover possible inaccuracies and weak spots of the measurements.

Dynamic light scattering (DLS) measurements remain crucial in the confirmation of the size distribution of nanoparticles in aqueous dispersion and their colloidal stability [43]. The determination of the hydrodynamic diameter (DH) is usually joined by the evaluation of the zeta potential representative of the surface charges since the change of this parameter is often used to confirm the surface modification of the nanomaterial [48,49]. However, with small biomolecules such as miRNA, the changes in DH and zeta potential are generally too small to be reliable proof of conjugation. Although DLS and zetametry measurements are very common and can be found in the majority of performed studies, they are also necessarily joined by other complementary techniques which allow for more accurate confirmations [50].

Gel electrophoresis (of both the agarose and the polyacrylamide types) can be used to determine whether the conjugation between the nanomaterial and the ligand, or between two ligands, occurred. The migration of the unattached ligands is noticeably different compared with the migration of the conjugated ones, allowing for the separation of both. As a control, samples of NPs conjugated to a nucleotide sequence complementary to target miRNA, the non-conjugated NPs [44,51], and the solutions of free sequences are often used [52,53]. However, when it comes to confirmation of the binding of a large number of biomolecules, it is necessary to have a reliable purification technique to eliminate the excess of free ligands.

Structural and elemental analyses with X-ray diffraction (XRD) and X-ray photoelectron spectroscopy are often combined [38] and are generally used as a precise way to study the composition and bulk properties of a nanomaterial, including the presence of biomolecules on its surface. XRD provides information about the structural properties of a nanomaterial [44], including crystallinity and phase, while it can also give a rough idea of the average size of the NPs. XPS is the most sensitive spatially resolved technique enabling the determination of the bonding nature and elemental ratio in the nanomaterial, data from which the composition of the layers can be deduced [35]. Both techniques are less efficient with very small NPs, with smaller precision in structural measurements, and with amorphous NPs, where different atomic lengths can affect the measurement.

Thermogravimetric analysis (TGA) is centered on the weight loss of a sample as a function of increased temperature. Due to the organic content, the characteristic weight loss profile before and after the conjugation is investigated [12,14]. Generally, TGA is useful to confirm the presence of organic molecules conjugated to the inorganic nanomaterial, as is also the case with spectroscopic techniques.

Fourier transform infrared (FT-IR) spectroscopy is based on the detection of certain functional groups present on the surface of the nanomaterial in the event of successful conjugation and is considered acceptable for the detection of biological ligands onto inorganic nanomaterial [34,54,55]. Nevertheless, FT-IR is not always sensitive enough. Additionally, non-reacted components need to be removed with the appropriate purification method prior to FT-IR measurements.

Fluorescence spectroscopy is quite favored for confirmation of both the successful functionalization of the nanomaterial and the subsequent hybridization of target miRNA. The strategies are based on either the quenching of the fluorescence signal [56] or its increase after the binding event [34]. It can be the nanomaterial itself that has fluorescent properties, such as QDs, or a complementary sequence to the target miRNA modified with fluorescent dye. Therefore, in the case of using fluorescent components, the characterization of the nanoprobe and the detection of the target miRNA capture are closely entwined. Notably, it is important to have an efficient strategy of sample purification or a reliable way to distinguish the signals, as the non-conjugated fluorescence components can easily interfere with the accuracy of the measurements.

## 3. Conjugation Strategies Leading to Functionalization

The coating of the nanomaterial, more specifically of nanoparticles, improves the chemical and colloidal stability, provides surface modification available for further functionalization, and can alter the properties of NPs [57].

Selection of the conjugation method is influenced by: (i) available reactive groups on the surface of the nanomaterial and on the ligands intended to be conjugated; (ii) chemical and mechanical stability of both precursors and final product; and (iii) simplicity and reproducibility of the synthetic procedure, along with its cost-effectiveness [58].

**Figure 2:**
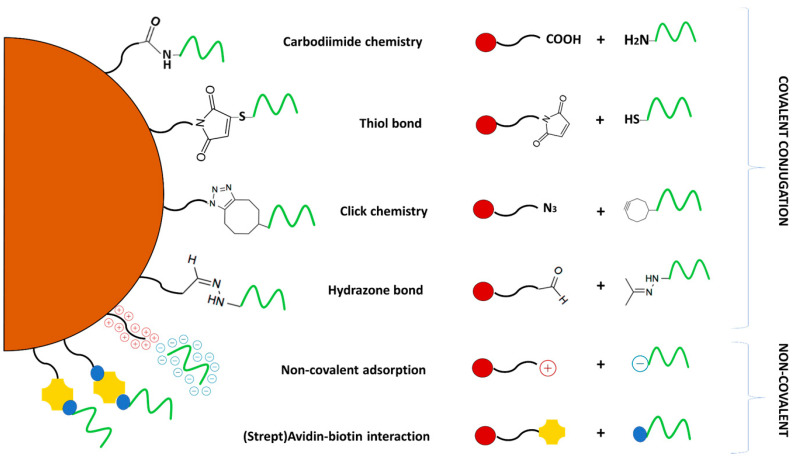
Schematic representations of covalent and non-covalent conjugation approaches.

Nanomaterials can be conjugated with various targeting biomolecules (such as proteins, nucleic acids, antibodies, and small drug molecules) via covalent or non-covalent assembly, which is more or less stable, respectively. Covalent conjugation is possible to achieve through the direct conjugation method, click chemistry, cross-linking strategies, etc. Non-covalent conjugation relies on physical interactions, such as hydrogen bonds, and interactions by electrostatic attraction or hydrophobic behavior. It should be noted that experimental approaches for formulating miRNA nanoprobes often combine covalent and non-covalent attachment to better exploit the advantages of both strategies, summarized in Table 2. Similarly, the most common approaches mentioned in the text are put into focus in Figure 2.

### 3.1. Covalent Conjugation

Covalent conjugation offers high stability and reaction efficiency. Several functional groups can form a covalent bond on the surface of nanomaterial and ligand, such as carboxyl (-COOH), amine (-NH_2_), aldehyde (-CHO), and thiol (-SH, sulfhydryl). In the case of the absence of a reactive group on some biomolecules and drugs, modification is possible before the conjugation [58]. The most popular covalent conjugations are carbodiimide chemistry and thiol bond, with widespread application.

#### 3.1.1. Carboxylic Acid–Amine Bond

Amines, a general label for compounds with a nitrogen atom with a free electron pair, are reactive due to their nucleophilic ability (donating the free electron pair to form a bond). This is especially the case for primary amines, making them ideal participants in conjugation with reactive groups. Amines are also widely used for the modification of biomolecules. For example, nearly all proteins have free amine at the N-terminus. The ending parts of proteins (C-terminus) often contain carboxyl groups which are also found on the side chains of amino acids (e.g., glutamate and aspartate). Carboxylic acids are very potent for fast reactions with nucleophilic compounds, where the hydrogen from acid is removed to form an anion, which is unsusceptible to additional reactions with a second nucleophilic compound [59].

One of the most widely used types of links is the amide bonds formed by the reaction of the amine group with N-hydroxysuccinimidyl (NHS)-activated carboxylic compound. In this method, the carboxylic compound has a first reaction with 1-ethyl-3-(3-dimethylaminopropyl)carbodiimide (EDC) and NHS to form an acyl amino ester, followed by a subsequent reaction with an amine to create the amide bond of exceptional stability. EDC is a universal cross-linking agent; however, after the reaction with the carboxyl group, some unstable intermediate is formed with a very short life in aqueous solutions, hence, the addition of NHS acts as a stabilizing agent [9].

In miRNA detection, NHS can be used for activation of carboxyl-modified nanomaterials, such as magnetic beads [60,61], and subsequent attachment of NH_2_-modified miRNA antisense strain. For example, the carboxylic acid group can be attached to the surface magnetic nanoparticles with silica coating [55]. Alternatively, a single-stranded DNA probe with COOH− group on one end can be covalently bound to amino-modified magnetic iron oxide nanoparticles [62].

A similar principle was utilized by Mahani et al., with a molecular beacon in a hairpin structure with a fluorescent quencher at one end and the NH_2_ group at the other. Carbon QD with the carboxyl groups on the surface formed an amide link with a molecular beacon, and only weak fluorescence was observed for the whole complex. In the presence of target miRNA-21, the hybridization of the hairpin structure led to the opening of the molecular beacon loop. The change in the distance between the quencher and the QD led to a change and increase of the fluorescent signal [63].

Amination, a reductive reaction where the amine group is combined with an organic molecule by transforming a carbonyl group (such as the carboxylic group), was used by Salimi et al. for the functionalization of graphene oxide. Graphene oxide is suitable for reduction and functionalization, and after functionalization with amine, the resulting graphene presents acceptable material for the attachment of biomolecules. Salimi et al. used this fact in the fabrication of an electrochemical biosensor for the selective detection of miRNA. Amino-functionalized reduced graphene oxide was assembled on the surface of a glassy carbon electrode with glutaraldehyde as a cross-linking agent, and a special sequence of miRNA-containing terminal amino group was attached for the detection of target miRNA [64].

Horny et al. utilized magnetic hyperthermia, with superparamagnetic core–shell γ-Fe_2_O_3_–SiO_2_ NPs working as nanoheaters. Maghemite cores were slightly pre-aggregated before coating with a silica shell, the elongated shape proposing suitable properties for magnetic hyperthermia. Since silica-coated core–shell nanoparticles with a maghemite γ-Fe_2_O_3_ core had -NH_2_ groups present on the surface, it allowed for the attachment of carboxyl-modified DNA probes [65].

Gao et al. combined carbon dots with graphene oxide. Carbon dots were linked with the amine-modified complementary DNA, and this complex was adsorbed on the surface of graphene oxide, quenching the fluorescence signal of the carbon dots. In the presence of target miRNA, the DNA desorbed from the surface of graphene oxide, hybridizing with the target miRNA and renewing the fluorescence signal, in proportion to the concentration of target miRNA [34].

Notably, amine modification of complementary oligonucleotide sequences continues to be the popular approach. Yao et al. conjugated iodide-modified magnetic beads with carboxyl groups on the surface and amine-modified DNA probes [42]. Xu et al. linked an amine-modified DNA probe with core–shell Au–Ag NPs covered with 5,5′-dithiobis (2-nitrobenzoic acid) [53]. Yazdanparast et al. synthesized magnetic core–shell Fe_3_O_4_–Ag NPs with carboxylic groups on the surface and joined them with amine-modified C-miRNA, applying the complex on a magnetic bar carbon paste electrode [54].

Carbodiimide chemistry is one of the most common approaches to binding miRNA to the nanomaterial. The reaction can be performed in an aqueous medium with no need for a complex solvent system, leading to a widespread application. However, the best efficiency is achieved in acidic pH, and it is necessary to perform it in buffers without additional amine and carboxylic groups. Depending on the nanomaterial, the challenge is presented in the form of the alignment of ligands and their binding orientation on the surface of the nanomaterial with multiple amine groups present. Moreover, the presence of additional amine and carboxylic groups (in ligands, on the nanomaterial, and in biological fluids) can also lead to a lower selectivity of the reaction [58,66].

#### 3.1.2. Thiol Bond

Thiol is a compound with the -SH pair linked to single carbon or a carbon-containing group of atoms. Thiols play a large role in biological systems, as they maintain suitable levels of oxidation and reduction state of cells, proteins, and organisms. It is also possible to link thiol and amine groups covalently (using a heterobifunctional coupling agent with one sulfhydryl-reactive and one amine-reactive group) or to link thiol and alcohol groups (similarly with an agent containing a hydroxyl reactive site). Alternatively, a biomolecule can be attached directly to the surface of the nanomaterial, using a dative (coordination) bond, generally formed by the biomolecule donating two electrons from a single atom. This usually happens with metal affinity coordination and thiol interactions. The formed bond is longer than the covalent bond, with higher energy and sensitivity to oxidation and change in pH. Metal-affinity bonds are formed between cationic metal on the surface of the nanomaterial and, for example, imidazole ring present at histidine residues. Affinity is increased with a larger number of histidine amino acids [9].

The high affinity of thiol groups for gold results in a self-assembled monolayer on the gold surface, or simply in the Au–S bond (which is more stable than a self-assembled monolayer), making it quite popular and gaining it widespread application. The strength of the bond between isolated thiols and gold depends on the properties of the gold surface, interaction time, and the pH of the solution. Coordinate Au–SH bond shifts to a covalent nature of the Au–S bond with higher pH. The reaction offers better efficiency at neutral pH and is suitable for nanomaterials or ligands that are less stable at acidic pH [67]. In many cases, thiolated capture probes with complementary sequences to target miRNA are used in some combination with AuNPs [21]. For example, Al Mubarak et al. covalently bound a hairpin probe of complementary DNA to the surface localized AuNPs on a gold substrate by using 1,4-benzenedithiol (a benzene core with two -SH groups in opposite places) [68].

M. Wang et al. obtained a strong electrochemical response of AgNPs in combination with AuNPs on a glassy carbon electrode for miRNA detection. AgNPs were adsorbed on the electrode subsequently after AuNPs with a clearly detectable silver peak. When AuNPs functionalized with thiolated complementary DNA were present on the electrode, steric hindrance and electrostatic repulsion prevented the adsorption of AgNPs, decreasing the signal response of AgNPs. In the presence of target miRNA, the complementary DNA was hybridized on the surface of AuNPs, forming a duplex. Duplex-specific nuclease, an enzyme able to disrupt the bond between the nucleotides in the duplex, leads to the elimination of the duplex from the surface of AuNPs. AgNPs could be then adsorbed on the electrode, providing detectable signal amplification and leading to the determination of the amount of target miRNA [39].

A similar approach of AuNPs (combined with polypyrrole-reduced graphene oxide) deposited on a glassy carbon electrode enabled the immobilization of thiolated capture probes, permitting the further formation of hairpin probes [52]. In the same vein, the self-assembly of thiolated capture probes and their subsequent immobilization on a pencil graphite electrode, modified with carbon black material and AuNPs, allowed the capture of the target miRNA [40].

A complementary DNA probe modified with SH can be covalently bonded to the AuNP surface [14,29,41], and similarly, the same type of modification can be used for a conjugation on AgNPs [18,69], core–shell Au–Ag NPs [26], Au–Ag nanocages [20], AuNP–peptide nanotube composites [12], Au-coated conglomerates of superparamagnetic NPs [43], citrate-capped AuNPs electrostatically adsorbed to Fe_3_O_4_ NPs [70], Ag nanoclusters on Au electrode [71], and hollow Au–Ag nanospheres [72].

The maleimide chemistry, along with the carbodiimide chemistry, is a very common strategy in miRNA adsorption to the nanomaterial. The functional groups of the reaction are less common in biological fluids, leading to better selectivity and ligand orientation. However, the thiolation of ligands can lead to a change in their chemical structure, negatively influencing the binding affinity. The presence of proteins containing thiol groups (such as in serums) can also lead to a less selective reaction [10].

#### 3.1.3. Click Chemistry and Hydrazone Bond

As miRNA capture and detection can still be considered a new branch of scientific research, many of the covalent conjugation strategies that are regularly used for binding targeting biomolecules or ligands to nanomaterials [9,10] have not yet been applied for miRNA, or only rarely. Two such examples are presented here, namely click chemistry and hydrazone bond.

In the variety of conjugation strategies with biomolecules exists the problem of undesired side reactions, due to the vast number of reactive sites and functional groups. While this is partially managed with specific coupling agents, they also may react with other functional groups (one such example is NHS, able to react with sulfhydryl and hydroxyl groups). This led to the development of bio-orthogonal chemistry, where reagents are stable (not prone to oxidization or hydrolyzation process) and have a reactive site that reacts only with a specific functional group.

These types of reactions are often referred to as click chemistry with their concept lying in a reaction that occurs quickly, under moderate conditions, and leads to a specific product (possibly with an easily removed by-product). Leading advancements of click chemistry were the developments of copper-catalyzed click chemistry followed by the copper-free approach, Diels–Alder cycloadditions, or Thiol–Michael click reactions. Click chemistry is also very popular in gene therapeutics, for easy conjugation of RNA, mRNA, and siRNA with, for example, fluorescent molecules [9,73].

Lu et al. used aggregation of AuNPs amplified by click chemistry for the detection of plant miRNA by DLS. Two probes, probe A, modified with dibenzocyclooctyne, and probe B, modified with azide, were synthesized to form a complementary sequence to target miRNA together. Hybridization with target miRNA leads to the close vicinity of both functional groups, forming a complex. Similarly, two types of AuNPs, with two sequences complementary to probe A and probe B, led to the respective attachment of both probes, and when in the presence of target miRNA, allowed the formation of bigger aggregates of AuNPs [74].

L. Zhou et al. employed click chemistry, i.e., the copper (I)-catalyzed azide–alkyne cycloaddition to combine two nucleic acid strands containing G-quadruplex DNA sequences. Alkyne-modified hairpin strand and azide-modified strand were bound by click chemistry reaction, forming complementary DNA capture probes, which were deposited on a Au electrode modified with fullerene nanoparticles [35].

Another type of bio-orthogonal conjugation method has a basis in reductive amination reaction (amine group is combined with organic molecule), where a hydrazone bond is formed by a reaction between aldehydes and hydrazide groups. The synthetic procedure is straightforward with good reproducibility, and the unique advantage is the pH sensitivity of the resulting bond—the bond is stable at neutral pH but becomes easily affected at acidic pH [58].

Ye Wang applied hydrazone coupling chemistry catalyzed by aniline to conjugate a specific hexahistidine peptide to amine-functionalized DNA, which led to this complex being self-assembled onto the hydrophilic surface of fluorescent quantum dots [75].

These strategies are less commonly applied for the nano-conjugation of complementary miRNA sequences to the nanomaterial, due to a relatively recent focus on the conjugation via click chemistry, combined with even more recent interest in miRNA-related functionalization. Nevertheless, there is significant potential for more studies in this field.

### 3.2. Non-Covalent Conjugation

The formation of macromolecules through non-covalent bonding is very common in nature, using for example, hydrophobic effects, coordination chemistry, hydrogen bonding, and dipole interactions (electrostatic and weaker dispersion forces). Along with covalent attachment, thiolated molecules can be also used for non-covalent conjugation (generally depending on the pH of the solvent). If the biomolecule has available thiol residue, the sulfhydryl group can form a dative bond on the surface of the nanomaterial.

The method of non-covalent bonding presents several advantages, such as fast formulation, avoidance of complex bonding, and the possibility of a quick release in target sites while minimizing cytotoxicity. This results in simple and cost-effective synthesis requiring fewer chemical reagents, allowing for industrial production and clinical applications. Nevertheless, compared with the covalent bonds, non-covalent linking is weaker and more prone to dissolution out of specific conditions. Often the stability of such bonds is weak in biofluids, presenting a challenge to overcome for in vivo applications.

#### 3.2.1. Non-Covalent Adsorption—Electrostatic and Hydrophobic/Hydrophilic Interactions

Electrostatic and hydrophobic/hydrophilic interactions are very well-known, their attractiveness being in the rapid binding process with no further chemical modification steps necessary. However, this type of passive adsorption results in a non-specific bond between the nanomaterial and the biomolecule, leading to reduced stability in biological fluids, and complicating the in vivo applications and the characterization of the functionalized nanomaterial.

Electrostatic interaction and subsequent adsorption on the surface of the nanomaterial originate from the attraction of two opposite charges. This interaction is non-specific, relying on the strength of the respective charges of the two interacting parts, and is susceptible to changes in experimental conditions (such as temperature, pH, and ionic strength). There is also a possibility of electrostatic complex dissociation due to the presence of other competing biomolecules and the following exchange [9].

As miRNA molecules are negatively charged, the high positive charge of polyethylenimine-capped AuNPs is suitable for the concentration of target miRNA-155. Hakimian et al. mixed the resulting complex with citrate-capped AuNPs covalently attached to a thiolated hairpin DNA probe. Both types of AuNPs formed cross-linking aggregates due to probe–target attachment, allowing detectable aggregation of AuNPs [49].

Ajgaonkar et al. used graphene quantum dots doped with nitrogen as a slightly positively charged nanoplatform, non-covalently bonding the single-stranded DNA capture probe. The electrostatic binding of the capture probe and the π−π stacking onto the surface of the graphene-QDs altered their intrinsic fluorescence [76].

Despite the potential of non-covalent adsorption, the application remains limited due to the challenging and often weak stability presented by such bonds.

#### 3.2.2. Streptavidin–Biotin

A well-known example of affinity interactions is the (strept)avidin–biotin interaction, which is irreversible and stable (unaffected by variations of temperature, pH, organic solvents, or denaturing agents). The natural high-affinity interaction occurring between (strept)avidin and biotin is similar to the interaction between enzyme and substrate (or receptor and ligand), and the resulting bond is approaching the strength of the covalent bonding.

Streptavidin is similar to avidin (glycoprotein consisting of four identical subunits), and as a biotin-binding protein, it possesses four binding sites to biotin, one for each subunit. Compared with avidin, it has a lower charge with a smaller possibility of electrostatic interaction with other biomolecules (or cell membranes), which also makes the protein less soluble in an aqueous medium. Furthermore, streptavidin is a non-glycosylated protein (without carbohydrate residues), making the likelihood of a non-specific bond to other molecules via carbohydrate receptors significantly smaller. It is possible to insert biotin (vitamin B7) into biomolecules without affecting their properties or activity. Carboxylic acid in this small molecule also allows covalent conjugation [77].

Both the nanomaterial and/or the biomolecule can be biotinylated, making it a very popular conjugation of choice. Likewise, streptavidin and biotin modifications are widely commercially available, allowing for easy use (one such example is pre-prepared streptavidin-modified magnetic nanoparticles, e.g., magnetic beads [78,79], QDs [75], biotin-modified RNA [13], or biotinylated DNA sequences [78]).

Streptavidin modification of NPs can be also performed with a cross-linking agent, such as presented by Chan et al. concerning silica-coated iron oxide NPs. After the amino-functionalization of the NPs, streptavidin was bonded to the surface with glutaraldehyde acting as a cross-linking agent. The biotinylated DNA was then linked to the streptavidin-coated NPs, and the resulting nanoprobes were used for the capture of a complex formed by target miRNA and molecular beacon containing target miRNA sequence [80].

In our previous publication, we modified the surface of PEGylated SPOINs with streptavidin. Biotin at the end of the PEG chain allowed close coverage of streptavidin, and the creation of the sufficient streptavidin layer of available biotin-binding spots enabled the functionalization with biotinylated complementary miRNA sequences, leading to the formation of nanoprobes with the ability to capture the target miRNA [50].

As was already mentioned, the combination of two or more conjugation approaches is very common. Cheng et al. deposited a thiolated hairpin probe on the surface of a gold electrode via the Au–S bond, and its loop structure was opened with the hybridization of target miRNA. The second hairpin probe modified with biotin lost the loop structure in the presence of the open first probe, leaving the biotinylated end to bind with AgNPs modified with streptavidin. The addition of more biotinylated AgNPs led to the aggregation of AgNPs and a stronger electrochemical signal [81].

A drawback in the extremely popular streptavidin–biotin conjugation is the large structure of the final complex, as streptavidin is a protein, and biotin itself is usually linked to the respective nanomaterial (or biomolecule) using a spacer (such as PEG chain) or cross-linking agent, adding to the final size and potentially affecting the binding rate [9].

**Table 2:** Covalent and non-covalent strategies for the functionalization of nanomaterials.

	Conjugation Strategy	Advantages	Disadvantages	References
Covalent conjugation	Carboxylic acid—amine bond	+ high stability; + high conjugation efficiency; + no chemical modification on the ligands	− low selectivity; − absence of control over ligand orientation; − reactions in acidic and water-free media	[42,54,65]
	Thiol bond	+ fast and selective reactions; + neutral pH media that avoid NP aggregation	− thiolation may hinder the biological activities of ligands; − non-selective in media containing thiol groups	[14,26,52]
	Click chemistry	+ site-specific conjugation; + higher yield; + control of ligand orientation; + mild conditions	− relatively new and more complicated than other methods; − possible toxicity with Cu(I) catalysts	[33,74]
	Hydrazone bond	+ stable at neutral pH	− easily affected by acidic pH	[75]
Non-covalent conjugation	Non-covalent adsorption: Electrostatic and hydrophobic/hydrophilic interactions	+ fast and easy formulation; + reduced toxicity due to high charges of NPs	− weak interactions and poor stability − harder to characterize	[49,76,82]
	(Strept)avidin–biotin	+ specific interaction + irreversible and stable + good orientation of conjugated ligands	− the large structure of the final complex − the potentially lower binding rate	[13,80,81]

## 4. Conclusions

Since the recognition of miRNAs as novel prognostic biomarkers, their accurate sensing and detection became crucial, especially in non-invasively obtained biofluids. As such, methods based on nanomaterials present several advantages stemming from unique properties (e.g., optical, magnetic, and electrochemical) depending on the chosen nanomaterial. Quite often, two or more types of nanomaterial can be combined in an effort to bring together several of their advantageous properties.

Covalent conjugations (carbodiimide bond, thiol bond, click chemistry, and hydrazone bond) form a stable covalent bond, while non-covalent conjugations (non-covalent adsorption, electrostatic, hydrophobic/hydrophobic interactions, and streptavidin–biotin interaction) rely on physical interactions between the ligand and the nanomaterial. Covalent conjugation often offers high stability, yield, and efficiency, at the cost of sensitivity to the medium of the reaction and pH, with, at times, lower selectivity. Similarly, non-covalent conjugation shows fast and easy formulation with the avoidance of complex chemical modification; however, the interactions are often weak and harder to characterize.

The mentioned limitations present interesting challenges in the functionalization of the nanomaterial, with the procedure generally expected to be simple, fast, and efficient. First, functionalization is often difficult to determine and requires a combination of two or more analytical techniques. Second, the overall stability and longevity of a nanoprobe or functionalized nanomaterial have to be controlled. Third, the binding affinity of the chosen nanoplatform connected to the subsequent miRNA capture, and its sensitivity to the media, pH, and composition needs to be better determined. On the other hand, the biomolecules naturally present in the biological samples can interfere with the functionalized nanoplatforms and lead to decreased selectivity and/or sensitivity. This issue depends on the experimental design of the capture and still needs to be studied in depth.

Considering the relatively young research field of miRNA detection, some of the conjugation approaches, which were successfully applied for ligands and biomolecules other than RNA sequences and their variations, have yet to be used. This fact leaves ample space for presenting novel applications on the road to functionalization with the goal of miRNA sensing.

## Data Availability

No new data were created or analyzed in this study. Data sharing is not applicable to this article.
